# nuPRISM: Microfluidic Genome-Wide Phenotypic Screening Platform for Cellular Nuclei

**DOI:** 10.1021/acscentsci.2c00836

**Published:** 2022-11-28

**Authors:** Abdalla M. Abdrabou, Bill T. V. Duong, Kangfu Chen, Randy Singh Atwal, Mahmoud Labib, Sichun Lin, Stephane Angers, Shana O. Kelley

**Affiliations:** † Department of Biochemistry and Molecular Genetics, Feinberg School of Medicine, Northwestern University, Chicago, Illinois 60611, United States; ‡ Department of Chemistry, Northwestern University, Evanston, Illinois 60611, United States; § Department of Biomedical Engineering, Northwestern University, Evanston, Illinois 60611, United States; ∥ Department of Pharmaceutical Sciences, Leslie Dan Faculty of Pharmacy, University of Toronto, Toronto, Ontario M5S 3M2, Canada; ⊥ Department of Biochemistry, Faculty of Medicine, University of Toronto, Toronto, Ontario M5S 1A8, Canada; # Terrence Donnelly Centre for Cellular and Biomolecular Research, University of Toronto, Toronto, Ontario M5S 3E1, Canada

## Abstract

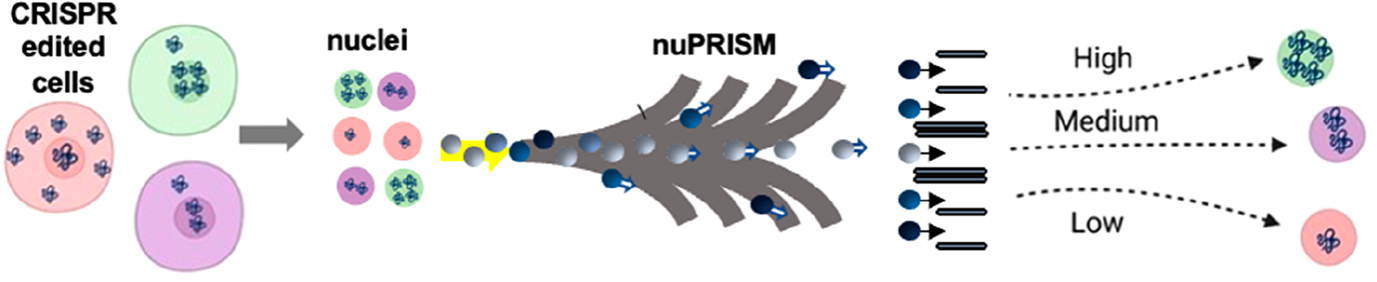

Genome-wide loss-of-function screens are critical tools to identify novel genetic regulators of intracellular proteins. However, studying the changes in the organelle-specific expression profile of intracellular proteins can be challenging due to protein localization differences across the whole cell, hindering context-dependent protein expression and activity analyses. Here, we describe nuPRISM, a microfluidics chip specifically designed for large-scale isolated nuclei sorting. The new device enables rapid genome-wide loss-of-function phenotypic CRISPR-Cas9 screens directed at intranuclear targets. We deployed this technology to identify novel genetic regulators of β-catenin nuclear accumulation, a phenotypic hallmark of *APC*-mutated colorectal cancer. nuPRISM expands our ability to capture aberrant nuclear morphological and functional traits associated with distinctive signal transduction and subcellular localization-driven functional processes with substantial resolution and high throughput.

## Abstract 2

nuPRISM is a high-throughput nuclear screening platform leveraging microfluidics and CRISPR genome-wide screens that identifies novel nuclear targets undetected by conventional platforms.

## Introduction

Advances in cell sorting methodologies have enabled multidimensional analysis of CRISPR-edited cell populations by integrating advanced sequencing, imaging, and cell sorting technologies through fluorescence-activated cell sorting (FACS)-based screens, single-cell functional genomics (scFG) screens, and arrayed screens using microscopy as a read-out.^1−3^ These approaches have significantly improved our understanding of phenotypic changes at the molecular level within multiple disease settings. Due to their broad applicability and ease of use in studying infection, cancer biology, and deciphering novel drug targets, genome-wide phenotypic CRISPR screens are continuously subjected to a myriad of modifications to boost their potential.^4−6^

Intracellular protein expression-based phenotypic screens are crucial in disease-biology research.^7−9^ However, studying the functional impact of a given protein of interest within a subcellular compartment, such as the nucleus or mitochondria, is often complicated by differences in subcellular localization-dependent protein activity as well as the background noise stemming from other biochemical processes the protein is involved in.^10−12^ Multifunctional intracellular proteins with protein activities across the whole cell are especially challenging and necessitate the development of sophisticated cell sorting strategies to delineate the functional relevance of the dynamic protein expression changes.

Conventional intracellular protein detection techniques, such as the immunoblotting and enzyme-linked immunosorbent assay (ELISA), have been developed to track intracellular proteins of therapeutic relevance across different subcellular compartments. However, these methods are hindered by multiple limitations in the study of dynamic protein translocations due to a complex workflow, the amount of sample required for robust signal detection, and the variability of experimental procedures across different settings that can affect biologically relevant phenotypic read-outs.^13,14^

High-throughput protein immunoassays that can be used as screening tools have addressed some of these limitations through robust quantifications and sensitivity, but the costly instrumentation, complex workflow variability, and applicability across a limited number of proteins have constrained their use.^15,16^ Moreover, the challenges related to the spectral imaging detection of specific proteins and specificity to antibodies, especially those expressed over multiple subcellular compartments, limit the use of flow cytometry or immunohistochemistry to precisely detect intracellular protein phenotypic changes.^17^

**Figure 1:**
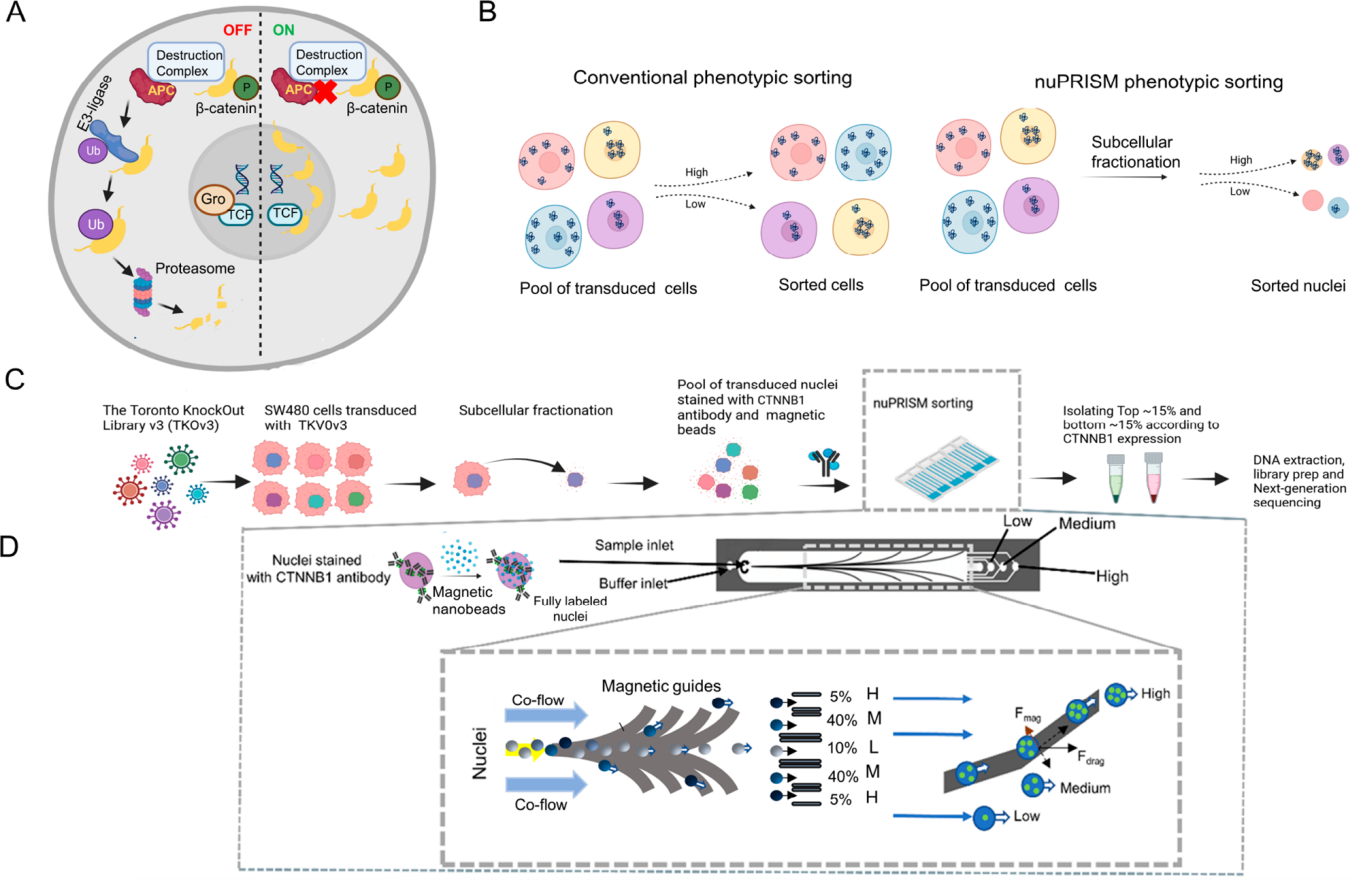
Overview of the nuPRISM workflow. (A) Illustration of the Wnt signaling pathway in both wildtype and mutant forms. (B) Schematic representation of the characteristic difference between conventional phenotypic screens and nuPRISM-based screens. (C) Schematic representation of the whole-genome CRISPR-editing and nuclei sorting-based screening assay. The cells were transduced with a genome-wide lentiviral library (TKOv3) at an M.O.I. of ∼0.25. After 14 days of doublings post-puromycin selection, the cells were subjected to subcellular fractionation, followed by staining with a β-catenin antibody and conjugating with 30 nm magnetic beads. (D) Schematic of the nuPRISM chip nuclei sorting principle, a derivation of the PRISM chip; the nuPRISM chip has a reduced channel height to allow high-throughput profiling of the isolated nuclei conjugated to magnetic beads under positive pressure flow (infusion mode).

An important disease-related phenotype that requires organelle-level analytical precision is the mutations of *adenomatous polyposis coli* (*APC*), which drive 80% of the sporadic cases of colorectal cancer. APC is part of the β-catenin destruction complex that targets β-catenin for proteasome-mediated degradation. In *APC*-mutated cells, β-catenin escapes degradation and accumulates in the nucleus, which leads to the activation of β-catenin target genes in a ligand-independent fashion, increasing the proliferation and invasiveness of the tumors (Figure 1A).^18−20^ Thus, identifying genes required for β-catenin nuclear localization would provide important insights into how this process is regulated and could contribute to developing more targeted and effective therapies.

While previous functional genomic screens have successfully identified genetic modulators of β-catenin expression across the whole cell in colorectal cancer model systems, there is still a lack of understanding about the aberrant reservoir of nuclear β-catenin and how best to target β-catenin nuclear shuttling and accumulation for potential therapeutic applications.^21,22^ Hence, there is a need for an approach that can profile cell nuclei according to β-catenin protein expression in a nondestructive manner that preserves the nuclei structure for subsequent downstream assays.

Advances in microfluidics have presented new opportunities to analyze biological processes with high throughput.^23^ For example, modifying the architecture of microfluidic devices to assess the deformability of cellular membranes has enabled targeted kinome-wide CRISPR screens.^24^ Moreover, the sensitivity of microfluidics permits efficient recovery of cells from the transient biological process for downstream analyses such as the ones involved in epigenetic regulation, detecting as low as 100 cells.^25^ Microfluidics has also been used to efficiently capture and detect rare cells such as the circulating tumor cells (CTCs) to enable more accurate characterization,^26^ as well as allowing ultrasensitive detection of surface proteins at single cell resolution within heterogeneous cell mixtures.^27^ Equally important, microscale technologies have enabled cell barcoding and sequencing providing a high-performance cellular characterization approach.^28^ Isotachophoresis is another capability that has allowed the isolation and purification of cytoplasmic nucleic acids at a single cell level.^29^ Thus, microfluidic immunomagnetic sorting is an attractive approach for further development.

Here, we describe nuPRISM—a microfluidics-driven functional genomics platform—as the first high-throughput cellular nuclei screening platform that enables the profiling of targeted protein expression with single nucleus resolution. As a proof-of-concept, we deployed the microfluidic nuPRISM chip to study levels of nuclear β-catenin accumulation in SW480 colorectal cancer cells. Through the use of whole-genome phenotypic CRISPR screening to discover genetic regulators of nuclear β-catenin levels, we identified and validated two novel positive regulators of β-catenin nuclear accumulation. The flexibility and high-throughput capacity of nuPRISM create a powerful tool to characterize nuclear phenotypes and track the subcellular localization differences of intranuclear markers at single-nucleus resolution.

## Design Overview

In normal colorectal cells, the destruction complex (consisting of Axin, APC, GSK-3a/b, CK1a) promotes β-catenin phosphorylation (Figure 1A), thereby targeting β-catenin ubiquitination by E3-ligase, which eventually marks β-catenin for proteasomal degradation. However, in *APC*-mutated cells, the process of β-catenin degradation is hindered, leading to constitutive activation of the Wnt signaling pathway, targeting β-catenin to the nucleus and contributing to invasiveness and proliferation seen in colorectal cancer cells. Blocking β-catenin nuclear localization by targeting regulators of this process may represent a novel and personalized therapeutic strategy for the treatment of colorectal cancers harboring *APC* mutation.

Conventional cell sorting-based CRISPR phenotypic screens have shown great promise in discovering novel modulators and functionally important intracellular protein targets. However, organelle-specific changes in protein subcellular localization are often difficult to track using conventional methods, creating a biological “blind spot” that can be overlooked. For example, *APC* mutations lead to nuclear accumulation of β-catenin to enhance signaling, but a substantial portion of the cytoplasmic β-catenin and the membrane-bound pools of β-catenin remain unchanged. As such, conventional cell sorting schemes would fail to detect phenotypic shifts in nuclear β-catenin levels.

Our nuPRISM platform overcomes this by sorting the nuclei post subcellular fractionation, enabling the detection of quantitative changes in the nuclear pool of β-catenin (Figure 1B). To perform a whole-genome phenotypic CRISPR screen, we first transduced the Toronto-Knockout Version 3 TKOv3 lentiviral CRISPR library at a low M.O.I. ∼ 0.25, followed by 48 h of puromycin selection. After 14 days, the cells were collected and subjected to subcellular fractionation to isolate the cellular nuclei. For each of the three replicates, ∼300 million nuclei were sorted at a flow rate of 2.5 mL h^–1^ using the nuPRISM chips. The enrichment of sgRNAs in each replicate was determined by NGS deep sequencing. In total, we processed ∼9 × 10^8^ nuclei on parallelized nuPRISM chips powered by syringe pumps. This arrayed setup enabled efficient sorting, requiring a total sorting time of around 9 h to process the entire whole-genome CRISPR screen.

The nuPRISM chip sorts nuclei based on the loading of iron oxide beads conjugated with a biotinylated antibody. The microfluidic chip architecture was optimized with reduced dimensions from our previous PRISM chip. This design allows us to obtain a high recovery rate of nuclei. Unlike other magnetic sorting systems like MACS, the recovery rate is increased given the high flow rate used that prevents nuclei from clumping. Also, the nuPRISM chip allows the separation of low, medium, and high populations, which is beneficial if the medium population is the target population or the application requires collection of a certain specific population instead of the positives and negatives. For nuclear β-catenin expression-based sorting, colorectal cancer SW480 cell nuclei were first labeled with biotinylated anti-β-catenin antibody and then conjugated with antibiotin magnetic nanobeads (Figure 1C). The magnetic labeled nuclei sample is then processed through a microfluidic chip. The magnetic guides deflect nuclei based on their β-catenin expression (Figure 1B). The lateral deflection of magnetically labeled nuclei along the magnetic beads is adjusted by balancing the hydrodynamic drag force and magnetic force the nuclei experience (Figure 1C). Cellular nuclei with higher β-catenin expression are expected to be loaded with higher amounts of magnetic nanobeads. Thus, nuclei with higher β-catenin expression were collected either in the medium outlet or high outlet, depending on the number of magnetic nanobeads bonded on the nuclei surface (Figure 1D).

To accommodate the application of cellular nuclei sorting, we configured the height of the flow channel in the nuPRISM chip to be 50 μm. Since a typical cell nucleus has a diameter of 6–10 μm, this optimized channel height prevents nuclei from clogging the flow channel and allows for high sample throughput while avoiding structural damage that can cause nuclei leakage. The optimized setup also enhances device sensitivity, enabling nuclei with low/no β-catenin expression to be easily collected in the low/zero outlet.

## Results

The immunomagnetic cellular nuclei sorting is facilitated by magnetic nanobeads conjugated to antibodies that bind to the intracellular protein target based on protein expression levels as validated by TEM (Supporting Information Figure 7). To enrich subpopulations of interest, we first optimized the flow rate to get the majority of the population expressing β-catenin to be collected in the medium outlet at ∼80% while retaining ∼10% in the high and the low outlets (Supporting Information Figure 2A,b). To evaluate the sensitivity of the nuPRISM method to detect population phenotypic drift, we transduced SW480 cells (exhibiting elevated levels of nuclear β-catenin accumulation) using four different single-guide RNAs (sgRNAs) targeting CTNNB1 and processed the CRISPR-edited pools of isolated SW480 cellular nuclei using the nuPRISM platform. In parallel, we also performed FACS-based nuclei sorting for a direct comparison of the two methods. Detection of β catenin^low^ cells was comparable between both methods, while the recovery using nuPRISM (∼85%) was considerably higher (Supporting Information Figure 2c). The higher recovery rate of the nuPRISM platform compared to FACS is mainly due to the ability to collect nuclei in Lo-bind tubes in real time rather than collecting them in FACS tubes then transferring again to Lo-bind tubes for subsequent processing. This significantly decreases recovery rates owing to the “sticky” nature of nuclei especially when collecting large populations as in CRISPR screens. Also, the microfluidic chips’ high flow rate, narrow dimensions, and preconditioning by 1% Pluronic acid prior to sorting prevents nonspecific nuclei attachment.

**Figure 2:**
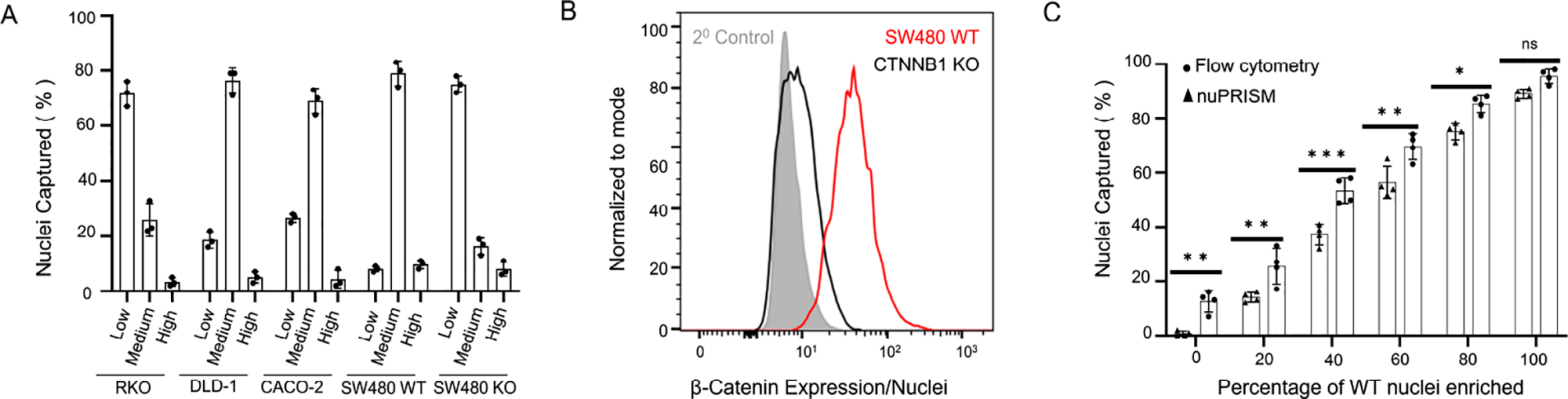
Assessing the nuPRISM capability to profile nuclei. (A) Enrichment profiles of nuclei isolated from a panel of colorectal cancer cell lines sorted with the nuPRISM chip. Isolated cellular nuclei were labeled with magnetic beads targeted to β-catenin and sorted at a flow rate of 2.5 mL h^–1^. Data are mean ± s.d. of *n* = 3 replicates. (B) Flow cytometry expression profiles show distributions of endogenous β-catenin protein levels in unmodified parental (SW480 WT) and CTNNB1-knockout populations. (C) Enrichment assessment across multiple predefined mixtures of wildtype and pooled β-catenin knockout (sgRNA #2) nuclei populations sorted using nuPRISM and FACS for comparison. Data are mean ± s.d. of *n* = 2 technical replicates. ***p < 0.001; **p < 0.01; * *p* < 0.05; NS: *p* > 0.05.

Based on the cell profiling capabilities of our existing microfluidic immunomagnetic cell sorting (MICS) platform, a high-throughput microfluidics platform that we previously used to perform a genome wide CRISPR screen in HAP1 cells to identify CD47 regulators.^4^ We designed the nuPRISM as a new microfluidic chip with optimized architectural parameters. These adjustments allow for high-speed profiling of isolated cellular nuclei based on the preferred intracellular target protein expression. We performed a series of tests to optimize the sorting rates and demonstrate the sensitivity of the new device to nuclear *β-catenin* expression changes upon transduction of the SW480 cells with sgRNAs targeting CTNNB1 (Figure 2C). We accurately recovered heterogeneous mixed populations of wildtype *β-catenin* and CTNNB1-knockout nuclei at a high flow rate (2.5 mL h^–1^). However, we have noticed the sensitivity of nuPRISM outperforms FACS at a higher CTNNB1 KO ratio and becomes more comparable as we increase the positive CTNNB1 population. This is mainly attributed to autofluorescence signals emitted by SW480 cells and the fixation and permeabilization reagents used prior to sorting to allow antibody intracellular staining. Since nuPRISM relies solely on magnetic beads and magnetic guide deflection, this issue is eliminated as it is unlikely that magnetic beads would bind to a nucleus with no protein expression as shown in TEM images (Supporting Information Figure 7). Moreover, we could recover the nuclei without structural damage to its membrane (Supporting Information Figure 3A,B), which can hinder the downstream processing.

To identify β-catenin nuclear regulators in *APC*-mutated colorectal cancer cells, we tested a panel of colorectal cancer cell lines to identify an ideal cell line for the whole-genome CRISPR screen (Figure 2A,B and Supporting Information Figure 3c). As expected, most of the nuclei from cells expressing wildtype *APC* ended up in the low outlet due to the low accumulation of endogenous nuclear β-catenin. On the contrary, nuclei of cells with *APC* mutation (SW480, DLD-1, and CACO-2) ended up in the medium and high outlets due to the higher level of nuclear β-catenin accumulation (Figure 2A,B and Supporting Information Figure 3C).

The SW480 cell line exhibited the optimum ratios of the high, medium, and low nuclear β-catenin expressing cells under basal conditions, ideal for identification of negative and positive regulators of β-catenin nuclear retention. To further assess the performance of the nuPRISM device, we mixed β-catenin wildtype and CTNNB1-knockout cells in predefined ratios to assess the profiling ability of the chip. Upon nuclei sorting, we observed accurate recovery that corresponded to the enriched population by flow cytometry and nuPRISM (Figure 2C).

Having established nuPRISM as a reliable cellular nucleus sorting strategy, we transduced the SW480 cells using the lentiviral TKOv3 CRISPR library for a genome-scale loss-of-function phenotypic CRISPR screen. The CRISPR-edited cell population was propagated for approximately seven doublings and was subsequently processed as three independent replicates for microfluidic sorting in parallel with FACS sorting. The isolated nuclei were sorted for the highest 15% and lowest 15% β*-*catenin expressing subpopulation enrichments.

The enrichment of sgRNAs in each of the nuclei subpopulations of interest was determined by the MAGeCK algorithm post-NGS deep sequencing. We also assessed the evenness of sgRNA reads across the three replicates in both high and low outlets of the chip using the Gini index (Supporting Information Figure 4B).^30^ The Gini index was below 0.2 in each, indicating an even distribution of sgRNA and a high-quality screen.

**Figure 3:**
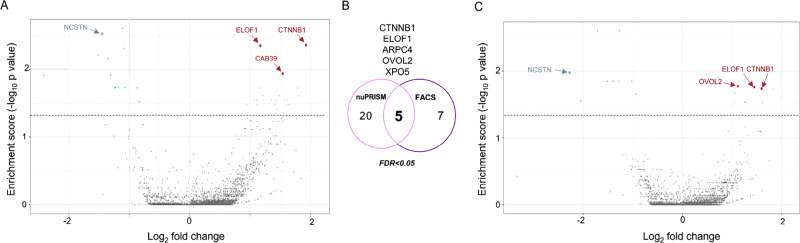
FACS and nuPRISM–CRISPR screens identify CAB38 & ELOF1 as modifiers of β-catenin. (A) Volcano plot depicting gRNA counts based on differential expression analysis in β-catenin^low^ versus β-catenin^high^ from NGS-seq results from post-nuPRISM sorting. (B) The overlap of hits from the nuPRISM and FACS β-catenin^ow^ screens at a false discovery rate (F.D.R.) of <5%. (C) Volcano plot depicting gRNA counts based differential expression analysis in β-catenin^low^ versus β-catenin^high^ from NGS-seq results from post FACS sorting.

To identify regulators of β-catenin nuclear accumulation, we compared the sgRNA read counts between the enriched and unsorted nuclei populations (Figure 3A,C). Both nuPRISM and the FACS-based sorting shared the top hit elongation factor 1 (ELOF1) and five hits in total with an FDR value below 5%, including CTNNB1. After the essential genes were excluded, the top hits from the nuPRISM screen are calcium-binding protein 39 (CAB39) and ELOF1. CAB39 is a calcium-binding protein that is implicated in hepatocellular and pancreatic carcinoma. It enables kinase binding activity to form STK11/STRAD complexes stimulating STK11 catalytic activity. ELOF1 is a small (10 kDa) zinc-finger protein that has a role in maintaining genome stability and directs RNA polymerase II ubiquitylation during transcription-coupled DNA repair. Both screens shared the top negative regulator gene *NCSTN* (Nicastrin). We performed gene set enrichment analysis (GSEA) using hits with an FDR < 30% to determine significantly enriched cellular pathways.

**Figure 4:**
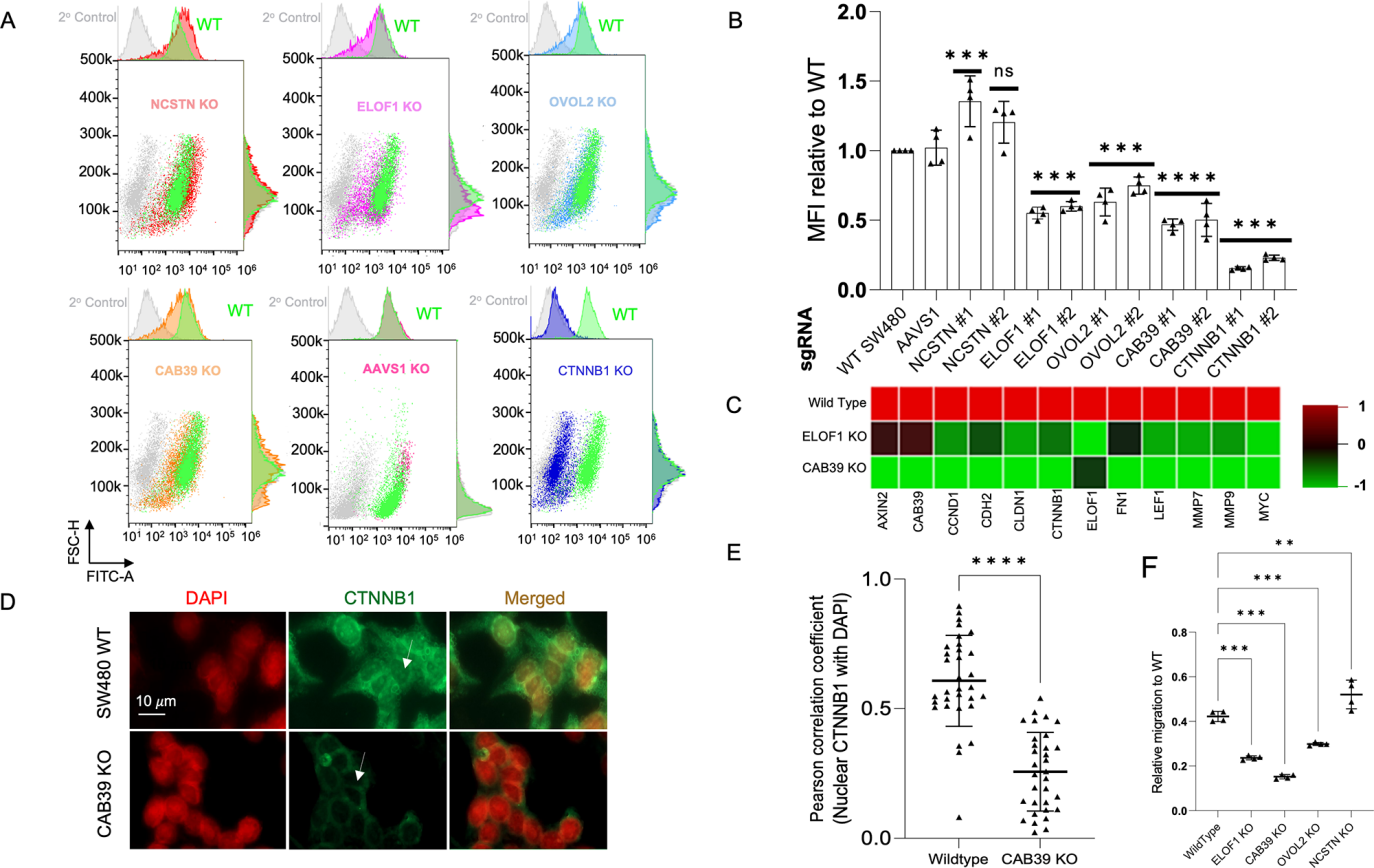
Validation of the screen hits on β-catenin nuclear retention. (A) Flow cytometry based β-catenin expression profiles of isolated nuclei populations from unmodified (WT) and SW480 cells transduced with sgRNAs targeting *NCSTN*, *ELOF1*, *OVOL2*, *CTNNB1*, *AVVS1*, or *CAB39* as indicated, 7–10 days after transduction. (B) Flow cytometry data from panel A data are represented as a bar graph of median fluorescence intensity relative to the wildtype of *n* ≥ 3 biological replicates from independent experiments. *p* values were calculated by two-way ANOVA, ***p < 0.001; **p < 0.01; *p < 0.05; NS: *p* > 0.05. (C) Heat map depicting an RT-qPCR based mRNA expression analysis of a panel of Wnt target genes in SW480 cell line transduced with either an sgRNA targeting ELOF1 or CAB39. All squares represent the mean of an independent experiment performed in technical triplicate. (D, E) Immunofluorescence intracellular staining imaging of SW480 CAB39 knockouts showing a decrease in nuclear retention of β-catenin (green). Nuclei were stained with DAPI (red). Pearson’s correlation coefficients for the colocalization of nuclear CTNNB1 and DAPI of WT SW480 cells or CAB39 KOs shown in panel D. (F) Bar graph comparing the number of WT SW480 cells versus a panel of K.O. cells (NCSTN, ELOF1, CAB39, and OVOL2) invaded across the Transwell membrane with or without the presence of the chemoattractant protein (FBS) in the culture media (*n* = 4 for each group).

Notably, the top process shows enrichment in pathways related to Wnt signaling pathways, such as degradation of Dvl and Axin, as well as essential pathways such as ribosome and cell cycle genes (Supporting Information Figure 6). To assess the impact on nuclear β-catenin accumulation, we designed a mini panel of sgRNAs targeting the candidate hits. We selected two sgRNA for each of the top positive regulators from the nuPRISM and the FACS screens as well as two sgRNAs targeting *NCSTN* as the top negative regular of β*-*catenin accumulation. We confirmed reduced nuclear β-catenin levels after transduction of sgRNAs other than the ones used in the screen targeting *ELOF1*, *CAB39*, and *OVOL2* as determined by intracellular flow cytometry and Western blotting (Figure 4A–C, Supplementary Figure 4a). As expected, the loss of NCSTN *expression* increased levels of nuclear β*-catenin* (Figure 4A).

Next, we performed RT-qPCR on Wnt target genes to assess the effect of loss of ELOF1 or CAB39 expression on the Wnt pathway (Figure 4D). Loss of CAB39 expression led to marked inhibition of expression of the Wnt pathway-related genes such as AXIN2, CCND1, CLDN1, CDH2, CTNNB1, MYC, LEF1, and MMP7, which is consistent with previous studies showing the association of the inhibition of these genes with decreased tumor progression and β-catenin nuclear retention.^31−33^ Furthermore, these results support the critical role of nuclear β-catenin in Wnt−β-catenin signaling in SW480 *APC*-mutant cells.

Loss of ELOF1 expression showed a similar profile as the CAB39 effect on Wnt target genes, but minimal or no change was observed in the expression of AXIN2. Using intracellular immunofluorescence, we also assessed the effect of loss of CAB39 expression on β-catenin nuclear accumulation and found a significant reduction of the nuclear β-catenin signal (Figure 4E,F). Finally, the interrogation of a panel of Wnt pathway related genes using RT-qPCR revealed several hits such as FN1, MMP7, and MMP9 that connected decreased migratory and invasive phenotypes observed in *SW480 ELOF1 KO* and *CAB39KO* cells to the epithelial-to-mesenchymal transition (EMT).

The invasive behavior of unmodified SW480 cells and CRISPR-edited SW480 cells with ELOF1 KO, CAB39 KO, OVOL2 KO, or NCSTN KO cells was tested using a Transwell cell invasion assay. A significantly higher number of NCSTN knockout cells migrated from the top to the bottom of the membrane compared to the control group (Figure 4G). Notably, the number of invading ELOF1 KO, CAB39 KO, and OVOL2 KO cells was less than control cells, suggesting nuclear β-catenin may have a role in regulating EMT remodeling. Accordingly, using RT-qPCR, we observed that the mRNA expression levels of the epithelial markers increased, while mesenchymal markers decreased when SW480 cells were transduced with sgRNAs targeting CAB39 and ELOF1 (Supporting Information Figure 5). Considering β-catenin’s role in the EMT process, these results further support the role of nuclear β-catenin accumulation in regulating this process.

Recently, it was reported that ELOF1 plays a crucial role in recruiting UV Stimulated Scaffold Protein A (UVSSA) to lesion-stalled Pol II. UVSSA recruits the deubiquitylating enzyme USP7, protecting CSB (Cockayne syndrome group B (CSB) protein) from proteasomal degradation mediated by the ubiquitin-selective segregase VCP (p97) (Supporting Information Figure 1A).^34^ Taking the positive role of USP7 in Wnt−β-catenin signaling-mediated growth of colorectal carcinoma cells into consideration, we hypothesized that this axis might play a role in ELOF1-mediated decrease in β-catenin nuclear accumulation.

**Figure 5:**
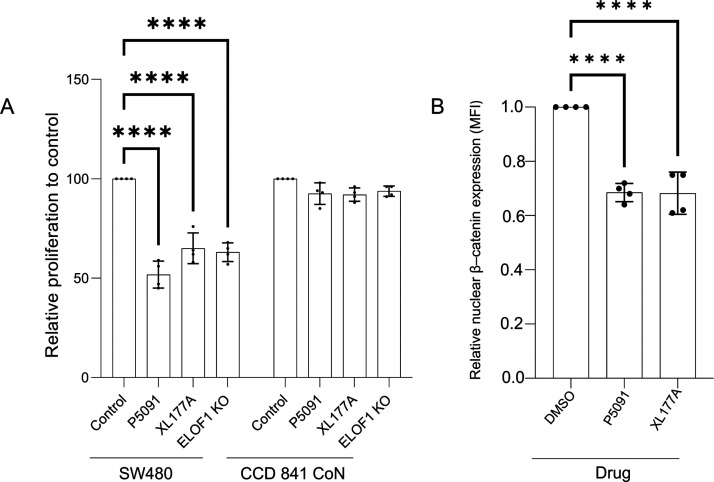
Druggability of the validated hits from the nuPRISM screen. (A) The effect of two different inhibitors and ELOF1 knockout on *APC*-mutated (SW480) and wildtype APC epithelial colorectal cell line (CCD 841 CoN) proliferation in a colony formation assay. Cells were seeded in 6 well plates and treated with DMSO or 5 μM of the indicated inhibitors. The drug-containing medium was refreshed every 24 h. Cells were fixed and stained with crystal violet when DMSO control wells reached confluency. (B), Intracellular flow cytometry (using β-catenin antibody) of nuclei isolated from SW480 cells after treatment with the inhibitors indicated (P5091 and XL177A) at a 5 μM concentration for 24 h.

Depletion of ELOF1 from SW480 cells decreased the levels of both USP7 and UVSSA (Supporting Information Figure 1B). Moreover, treating cells with USP7 inhibitors (XL177A and P5091) significantly affected cell growth and β-catenin nuclear accumulation (Figure 5B). We performed a colony formation assay to directly assess the growth inhibitory effect of P5091, XL177A, and ELOF1 KO in both SW480-*APC*-mutant colorectal cancer cells and normal colorectal cells. As shown in Figure 5A, both XL177A and P5091 exhibited a more substantial growth inhibition effect on the *APC*-mutated SW480 colorectal carcinoma cell line than normal colonic epithelial cell lines.

Nevertheless, both XL177A and P5091 treatments (5 μM) decreased the accumulation of nuclear β-catenin as determined by the intracellular flow cytometry staining in SW480 cells (Figure 5B). Also, the mRNA expression levels detected by RT-qPCR of UVSSA, USP7, and ELOF1 transcripts decreased significantly after treatment with XL177A or P5091 compared with the DMSO control group (Supporting Information Figure 1c). These results suggest that the ELOF1-UVSSA-USP7-nuclear β-catenin axis was partly involved in the observed cytotoxic activity of XL177A and P5091.

## Discussion

Genome-wide phenotypic CRISPR screens are typically used to isolate whole cell populations with either high or low expression levels of a given target of interest, analyze sgRNA enrichments across subpopulations of interest, and identify genetic modulators that regulate the expression of the protein of interest. To date, no platform has been explicitly adopted for cellular nuclei screening. Conventional phenotypic screens sort cells based on total protein expression across the entire cell, irrespective of the organelle-specific differences in protein expression and/or localization. This significantly impacts the accuracy and the specificity of the hits as the majority of multifunctional proteins usually exhibit differential subcellular localization across different organelles that corresponds to specific signaling outcomes and/or protein activity.

Our nuPRISM platform enables the discovery of targets regulating protein expression within the nucleus instead of the whole cell. High-throughput cellular nuclei sorting was applied to investigate the nuclear accumulation of β-catenin in *APC*-mutant colorectal cancer cells. Nuclear β-catenin accumulation in *APC*-mutant colorectal cancers is therapeutically relevant given its central role in Wnt signaling. Normally, cytoplasmic β-catenin is actively degraded upon interaction with the destruction complex (formed mainly by the three proteins APC, Axin, and GSK3a/β) and maintained at a low level without a Wnt ligand. However, in *APC*-mutant cells, the aberrant signaling causes β-catenin to escape degradation routes and accumulate in the nucleus. This shuttling activates specific Wnt target genes in parallel with the T-cell factor/lymphoid enhancer factor (TCF/LEF) family of transcription factors implicated in tumor relapse and resistance due to their functional role in colorectal cancer cancer-initiating cell survival.^19^ Moreover, this accumulation has been shown to cause radioresistance in colorectal cancer stem cells correlating with higher Wnt activation.^35^ These lines of evidence postulate the possibility of using β-catenin nuclear accumulation as a prognostic marker for CRC prognosis as it has a significant role in colorectal cancer progression.

Despite the compelling evidence supporting *APC* gene mutation as the primary regulator of β-catenin nuclear translocation, there is a lack of consensus that it is the sole justification for the heterogeneous nuclear accumulation and localization in the examined samples of *APC*-mutant tumors. Some additional proposed mechanisms involve the extracellular matrix through activation of integrin-associated kinases, which causes repression of E-cadherin and ultimately the nuclear localization of β-catenin that eventually dictates the behavior of the colon cancer cell.^36−38^ Also, trefoil factors have been implicated in the nuclear shuttling of β-catenin in colorectal cancer irrespective of *APC*-mutation status.^39^ Another study suggests the CRM1 mediated pathway is an additional independent nuclear transport mechanism of β-catenin.^40^

In the proof-of-concept whole-genome phenotypic CRISPR screen, we identified and validated two novel candidates (ELOF1 and CAB39) that showed a significant reduction of nuclear β-catenin accumulation. Interestingly, upon the knockout of these genes, we observed decreased expression of major Wnt signaling pathways related to genes such as CLDN1, CCND1, CDH2, CTNNB1, LEF1, MYC, and MMP7. All of these genes have been previously implicated as targets of the Wnt signaling pathway in colorectal cancer cells, and their reduced expression is usually associated with reduced cancer cell growth rate and proliferation. Thus, their decreased expression strengthens the role of our candidate hits in suppressing the aberrant phenotype in *APC*-mutant colorectal cancer cells. Notably, loss of ELOF1 expression did not affect the levels of AXIN2.

We also observed a marked shift in the expression of EMT process genes, consistent with the invasion and metastasis of adenocarcinomas that frequently arise from the loss of epithelial characteristics and gain of mesenchymal capacities of colorectal tumor cells. Accordingly, we saw a change in the number of migrating cells when we performed the Matrigel assay.

The Matrigel invasion assay showed a significant increase in SW480 NCSTN KO cells with corresponding higher levels of nuclear β-catenin, while a lower number of migrating cells were observed for SW480 ELOF1KO, CAB39 KO, or OVOL2 KO cells, all of which exhibit lower expression levels of nuclear β-catenin. Combined with a significant increase in epithelial markers such as *Desmoglein, alphaE*-catenin, Collagen alpha-1(IV), and a decrease in mesenchymal markers such as *Slug,* Snail, Vimentin, Mucin 1, Zinc finger E-box-binding homeobox 1 (ZEB1), these results demonstrate a loss of epithelial and gain of mesenchymal characteristics which is a known feature of the *APC*-mutant tumor cells. ELOF1 function in transcription-coupled nucleotide excision repair (TC-NER) is to promote the recruitment of the TC-NER factors UVSSA and TFIIH to repair transcription-blocking lesions. UVSSA recruits the deubiquitylating enzyme USP7, protecting CSB from proteasomal degradation mediated by the ubiquitin-selective segregase VCP (p97).^34^

Moreover, it has been shown that USP7 acts as a Wnt signaling inhibitor in *APC*-mutated colorectal cancers by restoring β-catenin ubiquitination, inhibiting tumor growth as evident in xenograft models growth suppression.^36,37^ We postulated that the ELOF1-UVSSA-USP7 axis is involved in the reduced β-catenin nuclear accumulation with ELOF1 being a regulator of the process. After we knocked out ELOF1, we saw decreased expressions of UVSSA, USP7, and CTNNB1. Moreover, targeting USP7 with small molecule inhibitors (XL177A and P5091) selectively inhibited the growth of mutant *APC*-colorectal cancer cells which are characterized by enhanced nuclear β-catenin accumulation but not wildtype *APC* epithelial colorectal cells. These results are consistent with the previous reports that suggest the role of USP7 in colorectal cancer is *APC*-mutation-dependent.^41,42^

Taken together, nuPRISM is a novel cellular nucleus sorting platform that merges advances in microfluidics technologies and phenotypic CRISPR screening to discover regulators of nuclear accumulation of proteins in a high-throughput manner. In our proof-of-concept nuclear β-catenin screen, we executed a genome-wide CRISPR phenotypic screen in under 9 h while maintaining the specificity of the nuclei’s antibody binding and nuclei structural integrity. We identified ELOF1 and CAB39 as novel nuclear β-catenin accumulation regulators and devised a novel sorting strategy that could be leveraged to target *APC*-mutant colorectal cancer. Overall, this study demonstrates the functionality and potential for nuPRISM to gain widespread implementation in the realm of drug discovery, especially for challenging intranuclear protein targets. The ability to profile the nuclear-specific functional phenotype of a protein at a single-nucleus resolution will open new avenues for targeted therapeutics development in colorectal cancer and other disease settings.

## Supporting Information Available

Supplemental figures and methods described through the manuscript (PDF)
sgRNA and primers list (XLSX)
Full list of CRISPR screen hits (XLSX)
