# Supplementary material for: nuPRISM: Microfluidic Genome-Wide Phenotypic Screening Platform for Cellular Nuclei
Source: ACS Cent Sci. 2022 Nov 28;8(12):1618–26. doi: 10.1021/acscentsci.2c00836 (PMC9801500; doi:10.1021/acscentsci.2c00836)
Supplement: Supplementary file 1 — oc2c00836_si_001.pdf [file oc2c00836_si_001.pdf]

nuPRISM: Microfluidic Genome-Wide Phenotypic Screening Platform for Cellular Nuclei

SUPPORTING INFORMATION & METHODS

Abdalla M. Abdrabou[1], Bill T.V. Duong[4], Kangfu Chen[4], Randy Singh Atwal[1], Mahmoud Labib[2], Sichun Lin[6], Stephane Angers[4,5,6,] and Shana O. Kelley[1,2,3,4]*

1. Department of Biochemistry and Molecular Genetics, Feinberg School of Medicine, Northwestern University, Chicago, Illinois, USA
2. Department of Chemistry, Northwestern University, Evanston, IL, USA
3. Department of Biomedical Engineering, Northwestern University, Evanston, IL, USA
4. Department of Pharmaceutical Sciences, Leslie Dan Faculty of Pharmacy, University of Toronto, Toronto, Ontario, Canada
5. Department of Biochemistry, Faculty of Medicine, University of Toronto, Toronto, Ontario, Canada
6. Terrence Donnelly Centre for Cellular and Biomolecular Research, University of Toronto, Toronto, Ontario, Canada

Contents

Supporting Figure 1: **Pathways involved in ELOF1 and CAB39 effect on β-catenin nuclear retention** S2

Supporting Figure 2: **nuPRISM chip optimization** S3

Supporting Figure 3: **nuPRISM chip array can sort and profile nuclei without structural damage** S4

Supporting Figure 4: **Quality control and assessment of genome-wide screen hits** S5

Supporting Figure 5: **RT-qPCR of a panel of E.M.T. genes in SW480 cell line** S6

Supporting Figure 6: **Gene set enrichment analysis of the screen hits** S7

Supporting Figure 7: **Retention of magnetic beads inside the nucleus** S8

Supporting Figure 8: **Gating strategy for flow cytometry experiments** S9

Methods S10

References S15

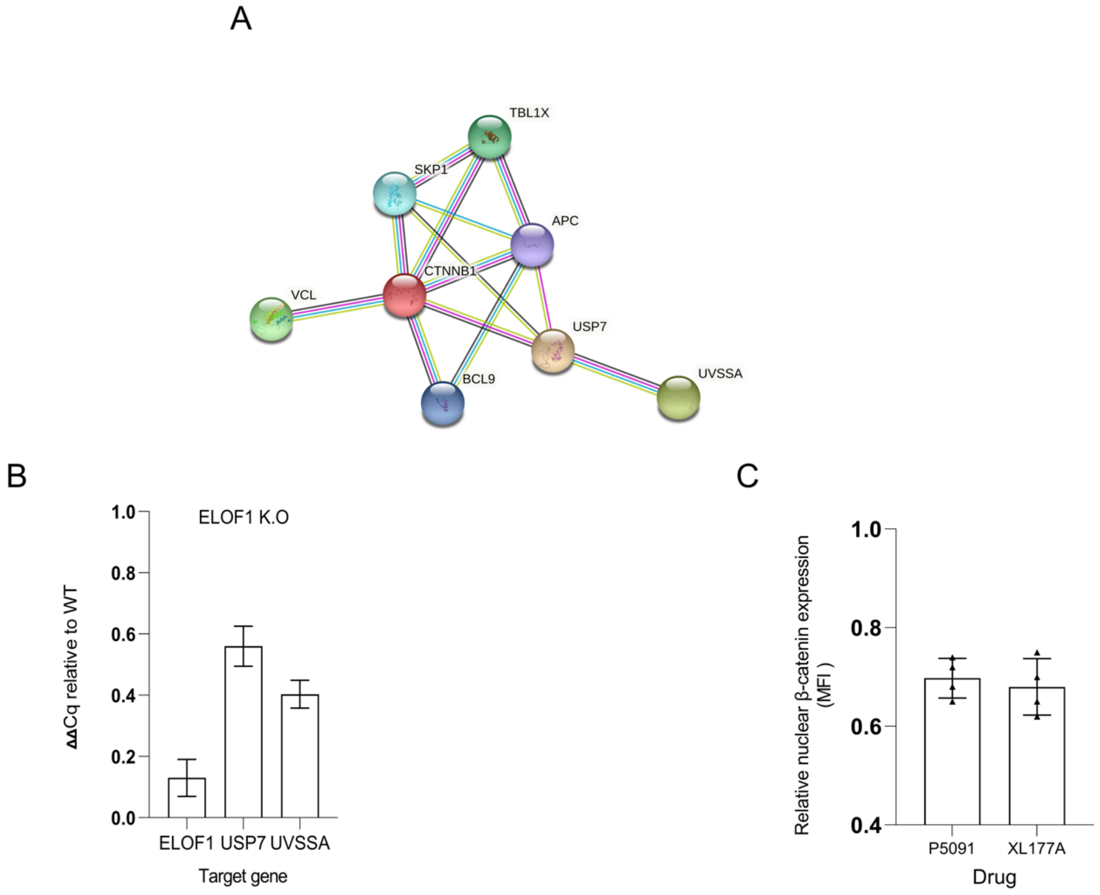

Supporting Figure 1. A, Interaction analysis of the ELOF1-USP7-UVSSA-β-catenin network using the STRING-db. **B**, RT-qPCR of 3 genes (ELOF1, USP7, and UVSSA) in SW480 cell line transduced with a sgRNA targeting ELOF1 gene. All bars represent the mean of an independent experiment performed in 3 technical triplicates. All bars represent mean ± s.e.m., *n* = 3 independent experiments unless otherwise noted. **C**, RT-qPCR of 4 genes (ELOF1, USP7, UVSSA, and CTNNB1) in SW480 cell line after 24 hours of treatment with the inhibitors indicated (P5091 and XL177A) at a 5uM concentration. All bars represent the mean of an independent experiment performed in 3 technical triplicates. All bars represent mean ± s.e.m., *n* = 3 independent experiments unless otherwise noted

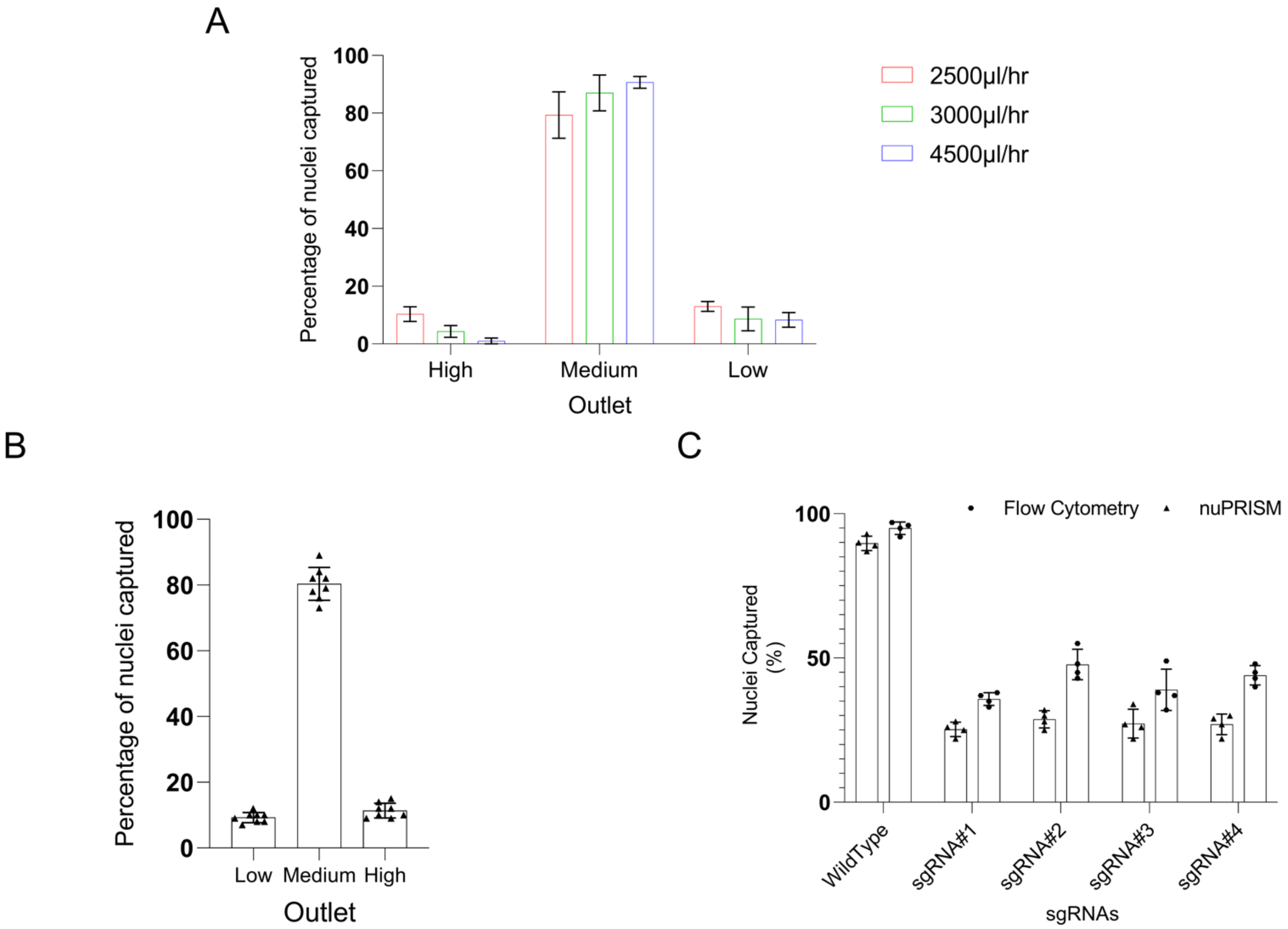

Supporting Figure 2. A, flow rate optimization to achieve ideal distributions into low/zero, medium and high outlets. SW480 nuclei were labelled with magnetic beads targeted to β-catenin. Data represent the mean ± SD of n=3 biological replicates. **B**, Outlet profile of SW480 nuclei sorted with the nuPRISM chip, labeled with magnetic beads targeted to β-catenin at a flow rate of 2.5 ml h^{-1}. Data are mean ± s.d. of *n* = 8 replicates. **C**, Comparison of the performance of nuPRISM chip and flow cytometry. The 4 sgRNAs from the TKVO3 library successfully generated β-catenin knockouts in the SW480 cell line. Data are mean ± s.d. of *n* = 2 technical replicates. Flow cytometry was performed on an aliquot of the same cell pool at 10d after transduction and subcellular fractionation to isolate the nuclei. β-catenin positive are the combined β-catenin ^{med} and β-catenin ^{high} populations. P values were calculated by two-way ANOVA, ***P<0.001; **P<0.01; *P<0.05; NS:P>0.05.

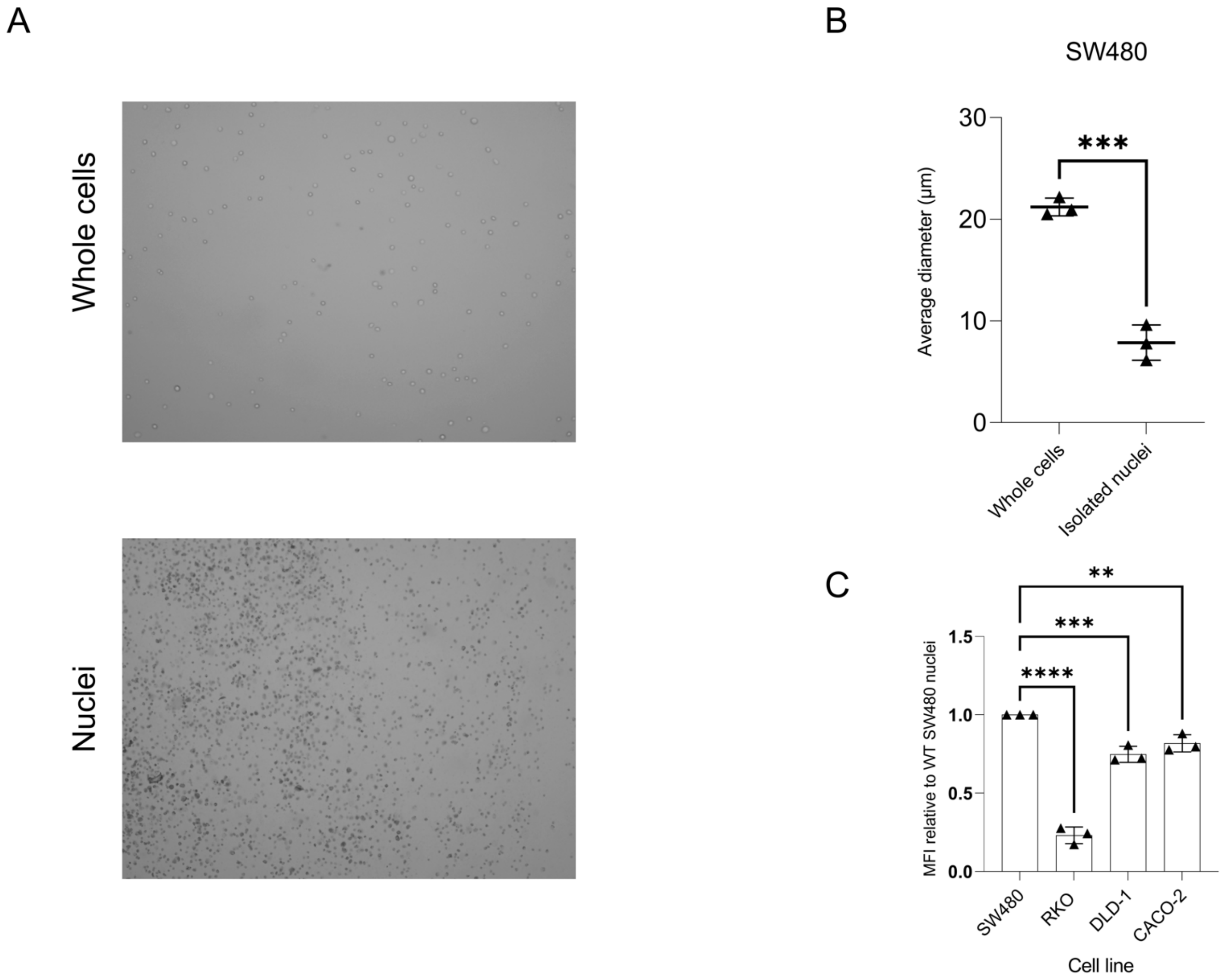

Supporting Figure 3. A, bright field images of SW480 nuclei post 2 rounds of sorting using the nuPRISM chips, and the right panel shows SW480 cells right after trypsinization and 3 washes of PBS. **B,** the cell size distribution of SW480 cells and SW480 nuclei counted on Countess Invitrogen II. The cells were counted after trypsinization and 3 PBS washes, the nuclei were counted after subcellular fractionation and before methanol fixation/permeabilization. Data are mean ± s.d. of $n = 4$ technical replicates. P values were calculated by unpaired t-test, ***P<0.001; **P<0.01; *P<0.05; NS:P>0.05. **C,** intracellular flow cytometry (β-catenin antibody) of nuclei isolated from a panel of wildtype colorectal cancer cell lines CACO-2, R.K.O., DLD-1, and SW480 cells to assess nuclear ***β***-catenin base levels. Data are represented as a bar graph as mean ± s.d. of the relative median fluorescence intensity (M.F.I., to wild type SW480) of $n = 3$ biological replicates from independent experiments.

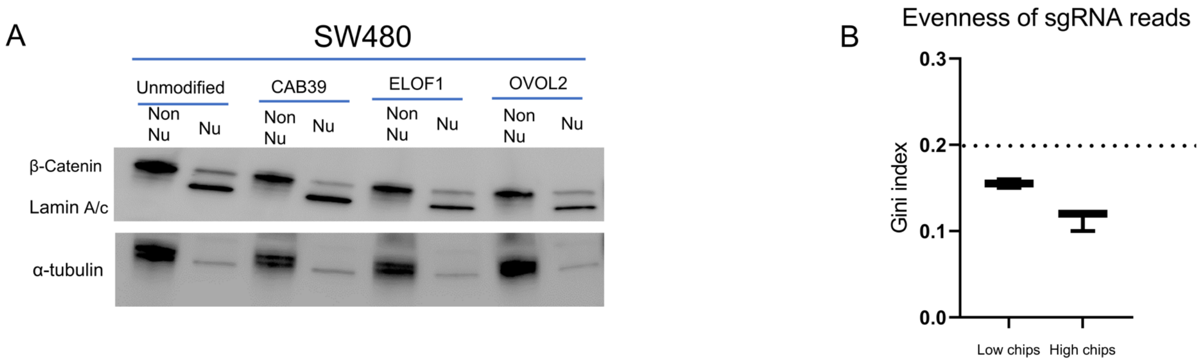

Supporting Figure 4. A, Immunoblots detecting β-Catenin protein nuclear and non-nuclear levels in control (WT SW480) and *ELOF1, CAB39 &OVOL2 knockouts*. **B**, A box and violin plot show the Gini index, a common measure of income inequality in economics, to measure the evenness of sgRNA read counts. A low Gini index cutoff is marked by a dashed line at <0.2. The screen results from the high and low nuPRISM enrichments suggest that the sgRNA read counts are distributed very homogenously across the target genes.

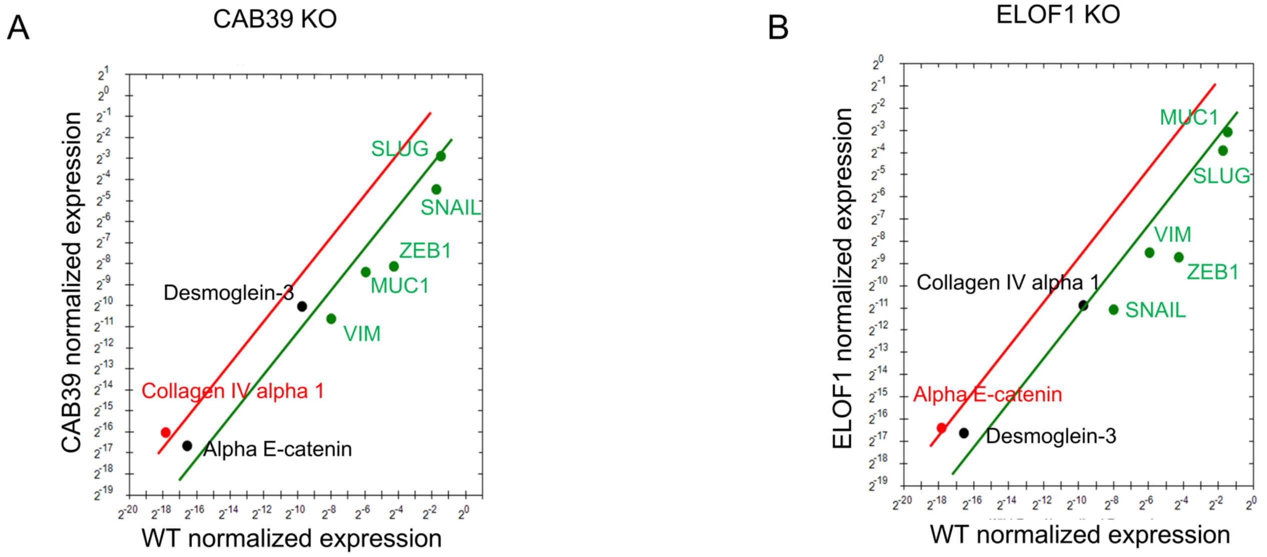

Supporting Figure 5. A, RT-qPCR of a panel of EMT. genes in CAB39 KO SW480 cell line. All points represent the mean of an independent experiment performed in 3 technical triplicates. **B**, RT-qPCR of a panel of E.M.T. genes in ELOF1 KO SW480 cell line. All points represent the mean of an independent experiment performed in 3 technical triplicates

A

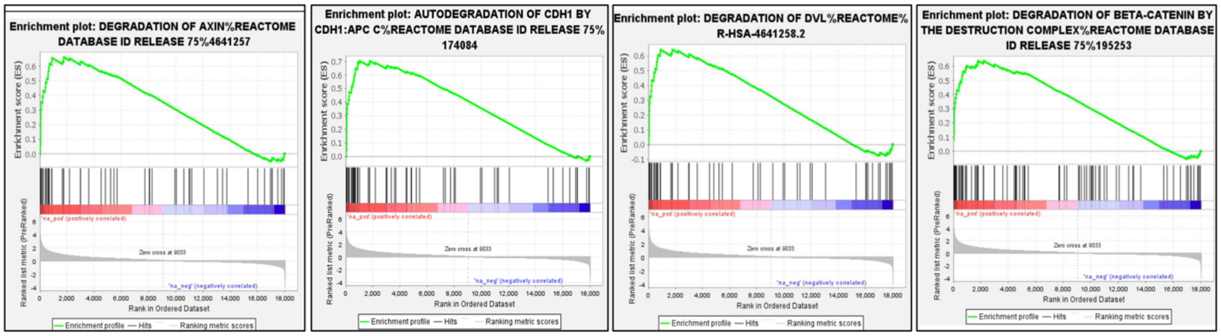

B

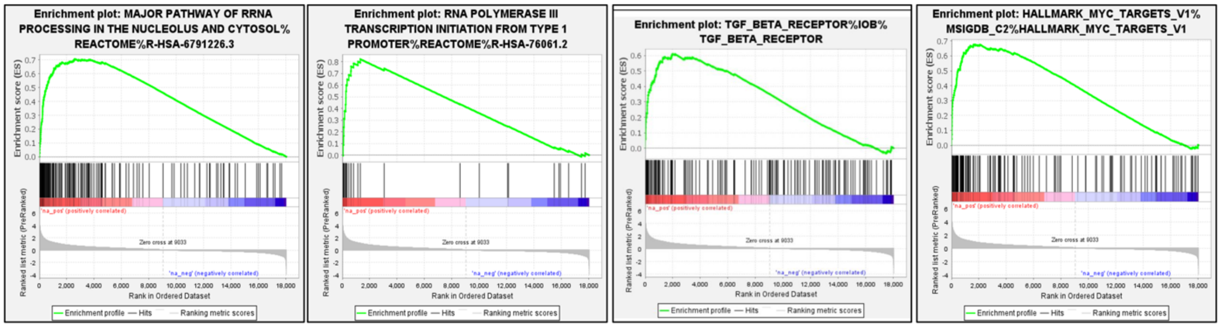

Supporting Figure 6. A-B, Gene set enrichment analysis (GSEA) of CRISPR screen hits with an FDR < 5%. The y-axis represents enrichment score (E.S.), and on the x-axis are genes (vertical black lines) represented in gene sets. The upper panel is for overall enrichments F.D.R. <5%, and the lower panel is for the top 4 GSEA related to WNT signaling pathway F.D.R. <5%.

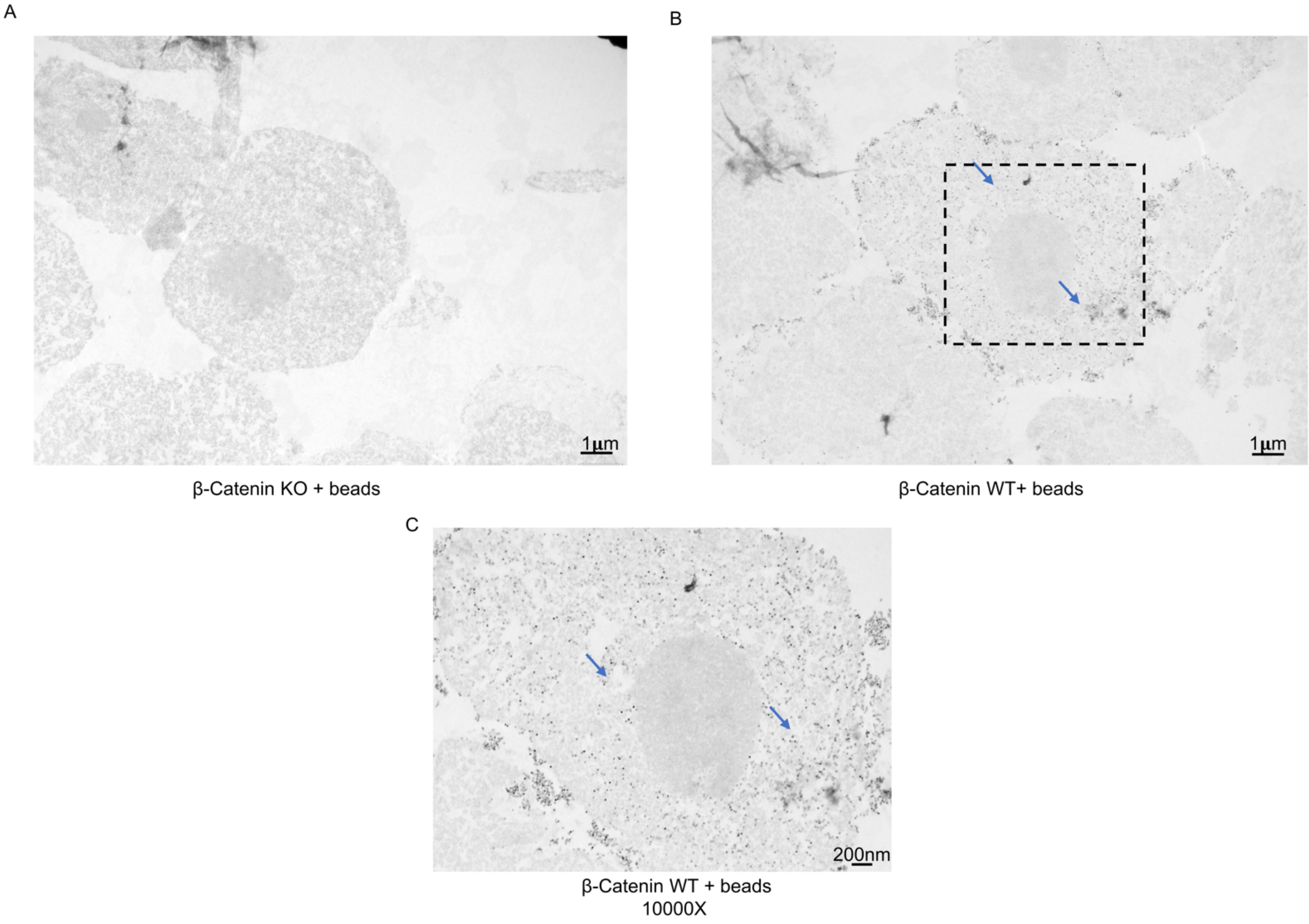

Supporting Figure 7. Retention of magnetic beads inside the nucleus. A-B, Representative Transmission electron microscopy (T.E.M.) images of SW480 nuclei, β-catenin wildtype (right image) and nuclei isolated from SW480 β-catenin K.O cell line (left image) after staining with biotinylated β -catenin antibody conjugated to magnetic beads with an avg size of 30nm and 6um for the nucleus as measured by the TEM. These are images of nuclei post subcellular fractionation. **C**, 1000x magnification of panel B.

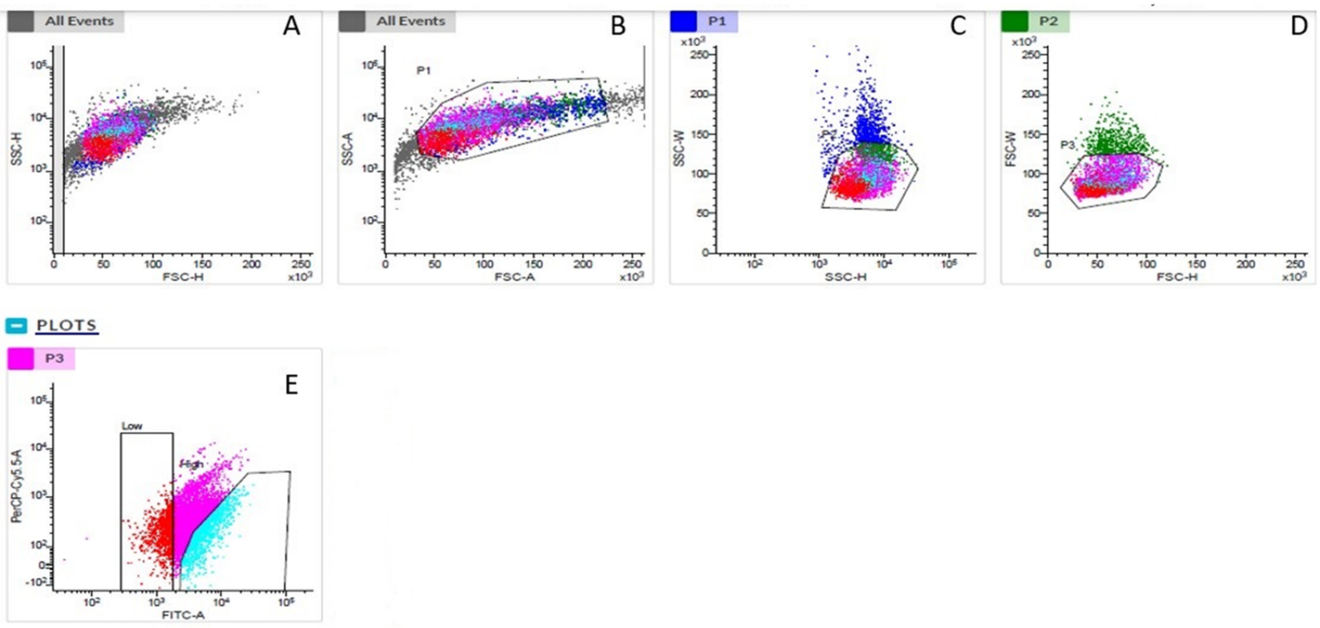

STATISTICS

Population	Events	% Parent	% Total	FSC-A Median	FSC-A %rCV	SSC-A Median	SSC-A %rCV
All Events	10,000		100.00 %	74264.81	45.68 %	6817.56	52.77 %
P1	8,610	86.10 %	86.10 %	73474.97	39.12 %	6736.40	43.55 %
P2	7,838	91.03 %	78.38 %	70239.48	35.19 %	6520.91	39.08 %
P3	7,237	92.33 %	72.37 %	67925.69	31.57 %	6314.22	35.84 %
High	1,187	16.40 %	11.87 %	70648.34	31.90 %	7014.65	34.05 %
Low	1,225	16.93 %	12.25 %	59073.95	22.03 %	4478.41	31.26 %

Supporting Figure 8. Gating strategy for flow cytometry experiments. A-B. Forward scatter area or height vs. side scatters area or height with gate 1 to separate cell events from debris. **C**. side scatter width vs. side scatter height to separate single cells from aggregates. **D**, forward scatter height vs. forward scatters width with gate 2 to separate single cells from aggregates. **E**, Gating for β-catenin -FITC conjugated antibody for the top 15% and bottom 15% expressing nuclei into their respective buckets.

Methods

Mammalian Cell Culture.

RKO, SW480, DLD-1, CCD-841 CoN and HEK-293T cells were obtained from ATCC. For screening, SW480 cells were cultured and maintained under standard conditions in DMEM medium (Gibco, Grand Island, New York, USA) containing 10% fetal bovine serum (Gibco) with and 1% penicillin–streptomycin (ThermoFisher). All the cells were cultured at 37 °C and 5% CO_2 in humidified incubators, were free of mycoplasma and routinely tested using the MycoAlert Detection Kit (Lonza).

MICS device fabrication

Metglas 2714A was first taped on a 100 mm glass wafer using epoxy and incubated under pressure for 24 hours. The metglas surface was then clean with acetone and isopropanol. Positive photoresist S1811 was then spin coated on the metglas surface. After incubation on a hotplate at 90 °C for 2 minutes, the glass wafer was placed on a mask aligner. The pattern of the magnetic guide from a chromium mask was transferred to the photoresist layer through photolithography. Then glass wafer was then developed in developer to remove exposed photoresist. After development, the glass wafer was bathed in an iron etchant consisting of 3.6% HCl (Sigma), 14.3% H2O2 (Sigma) and 82.1% H2O. The pattern was transferred from the photoresist layer to the metglas surface of the glass wafer after wet etching. The unexposed photoresist on the glass wafer was stripped off and the wafer was rinsed with isopropanol. The glass wafer with magnetic guide was encapsulated with a thin layer (16 µm) of SU8 3010 photoresist. The encapsulation layer of SU8 photoresist formed the bottom of the flow channel of the final chip. Another layer of SU8 3050 photoresist (50 µm) was then spin coated on glass wafer. The flow channel was patterned on the SU8 3050 through photolithography. The SU8 channel was bonded with a PDMS cover containing inlet/outlets ports as previously reported (1). Briefly, the PDMS cover was treated oxygen plasma and then bathed in 10% APTES solution (sigma) for 30 minutes. After rinsing with DI water, the PDMS cover was aligned and bonded with the SU8 channel. The bonded chip was incubated under pressure at 70 °C for 12 hours before usage.

Screen data processing and quality control

The MAGeCK algorithm is created to identify positively and negatively selected sgRNAs and genes in genome-scale CRISPR/Cas9 knockout experiments (4). It consists of several steps: read count normalization, mean-variance modelling, sgRNA ranking followed by gene ranking. These steps were executed as described in (). Briefly, we used the MAGeCK “count” command to generate read counts of all samples. We next used MAGeCK “test” command to identify the top negatively and positively selected sgRNAs. Finally, MAGeCK Algorithm determine for each gene whether its sgRNAs were enriched towards the top of the result list. The resulting enrichment *P* values were corrected for multiple testing using the Benjamini–Hochberg correction, resulting in a false-discovery rate (FDR)-corrected value The hits generated are provided in the supplementary table (?) for both FACS and nuPRISM. We assessed the quality by determining the number of missing sgRNAs and the Gini index. The Gini index, a popular measure of income inequality in economics to measure the evenness of sgRNA read counts. A high Gini index > 0.2 suggests that the sgRNA read count is distributed heterogeneously across the target genes. This could potentially be caused by multiple reasons such as unevenness in CRISPR oligonucleotide synthesis, or poor efficiency in viral transduction.

CRISPR sgRNA lentivirus production

The pLCKO-TKOv3 plasmid library lentivirus was produced as previously described (4). In brief, HEK293T cells were seeded at a density of 9×10^6 cells per 15 cm plate and incubated overnight, after which cells were transfected with a mixture of psPAX2 (4.8 µg; Addgene, 12260), pMDG.2 (3.2 µg; Addgene, 12259), TKOv3 plasmid library (8 µg) and X-tremeGENE 9 (48 µl; Roche) in Opti-MEM (Gibco). Then, 24 h after transfection, the medium was changed to DMEM with 1% BSA (Sigma) and 1% penicillin–streptomycin (Gibco). Virus-containing medium was collected 48 h after transfection, centrifuged at 1,500 r.p.m. for 5 min and stored at −80 °C. Functional titers were determined by virus titration on SW480 cells. Subsequently, 24 h after infection, the medium was replaced with puromycin-containing medium (1 µg ml^{-1}) and the cells were incubated for 48 h. The multiplicity of infection (MOI) was determined 72 h after infection by comparing survival of infected cells with infected unselected and uninfected selected control cells. Lentivirus for individual sgRNA constructs was produced on a smaller scale; HEK293T cells were seeded at a density of 0.5×10^6 per 6-well in low-antibiotic growth medium (DMEM with 10% FBS (Gibco), 0.1% penicillin–streptomycin) and incubated overnight.

Generation of *CTNNB1*, *ELOF1, CAB39, NCSTN* and *OVOL2* knockout cells

For transduction experiments using cell pools, vectors containing the sgRNA sequence were purchased from Genscript. RKO, CACO-2, CCD-841-CoN, HEK293T or SW480 cells were infected with target and control sgRNAs (MOI < 1) in the presence of 8 µg ml^{-1} polybrene. Then, 24 h after infection, the medium was replaced with fresh medium containing puromycin and cells were incubated for 48 h. Cells were further cultured in selection-free medium as indicated for individual experiments and were passaged every 2–4 d. An sgRNA targeting the *AAVS1* locus was used as negative control.

MICS sorting

SW480 cells were detached using 0.125% trypsin, washed once in PBS resuspended at a concentration of 1×10^7 cells ml^{-1} in a solution of PBS supplemented with 3% BSA. The cells were then subjected to subcellular fractionation as described below to isolate the nuclei. Throughout the sorting or isolation, the nuclei were processed in DNA LoBind® Tubes, (1.5ml, 15ml or 50ml), Catalog No. 0030122348, 0030122208 or 022431021 to obtain the most recovery possible. Nuclei were fixed and permeabilized by adding 95% (v/v) ice cold methanol to the nuclei pellet and incubating on ice for 30 min. Next, Nuclei were labelled for CTNNB1 expression with biotin anti-human CTNNB1 antibody (**β**-Catenin (D10A8) XP® Rabbit mAb (Biotinylated)(13727S) at 1 µl per 5 million nuclei. Excess antibody was removed by washing 3x in PBS with 3% BSA. Nuclei were resuspended at 1×10^7 nuclei ml^{-1}, and anti-Biotin Streptavidin Iron Oxide 30nm Nanoparticles were added at a 20% concentration by volume (Ocean Nanotech, SHS30-01) and incubated at room temperature for 60 min.

The chips were first degassed with water containing 1% w/v Pluronic F108 (BASF) and incubated for 15 minutes. Then the chips were washed with 3% BSA and incubated for 15 minutes to prevent nonspecific nuclei adhesion. 1 mL of 3% BSA was then loaded in the buffer reservoir as the co-flow buffer, and nuclei sample was loaded in the sample reservoir for processing. To obtain a high throughput while keeping high deflection efficiency, the sample withdraw flow rate was optimized to be 2.5 mL/hr. SW480 nuclei were sorted by nuPRISM at a concentration of around 10×10^6 cells ml^{-1} in PBS supplemented with 3%BSA. Syringe pumps (Fusion 200, Chemyx), operating in withdrawal mode, were used to drive flow in the MICS chips. Custom 3D-printed mounting hardware enabled up to five stacks of three syringes (containing 20 ml, 10 ml, and 3 ml syringes; Becton Dickinson) to be driven by the same pump. The different cross-sectional areas of the syringes were used to generate different flow rates, corresponding to the width of the low, medium, and high outlet channels. Two inlet reservoirs, one containing the sample (nuclei) solution and one containing a flow focusing buffer

stream (PBS with 3% BSA), were connected to the two inlets of the nuPRISM chip. The pump flow rate was chosen such that the sample flow rate was 2.5 ml h^{-1} unless indicated otherwise. Two wafers were used per replicate, where each wafer has 6 chips, and each chip can sort the nuclei (10 x 10^6) at a flow rate of 2.5 ml $^{-1}$. The sorted samples were collected in their respective syringes, and the volume of solution collected in each syringe was measured by weight. A small fraction (100 μl) of each sample was collected for cell counting to determine cell concentration after sorting. Recovery efficiency was defined as the percentage of input cells that were recovered in collected outlet populations. For the screen, the fractions collected from the low/ and high outlets were collected in 15 ml DNA LoBind® Tubes. Nuclei were then centrifuged and processed for genomic DNA extraction or frozen for downstream experiments.

Pooled genome wide CRISPR screens in SW480 cells

CRISPR screens in SW480 cells were performed essentially as previously described (2). In brief, 250 × 10^6 cells were infected with the TKOv3 lentiviral library at an MOI of around 0.25 (more than 500-fold coverage of the library after selection with puromycin). Medium was changed 24 h after infection to puromycin-containing medium (1 μg ml^{-1}). Then, 48 h after infection, 100 × 10^6 puromycin-selected cells were cryo-banked, 120 × 10^6 cells were split into three replicates of 40 × 10^6 cells, passaged every 2–4 d and maintained at 500-fold coverage. Subsequently, 40 × 10^6 cells were collected for genomic DNA extraction at T_0 after selection and at every passage until day 16 after selection (T_{16}), when sorting was performed. The unsorted T_{16} sample was used as a reference. Genomic DNA extraction, library preparation and sequencing were performed as described below.

Transwell migration assay

To prepare the migration assay chambers, 750 μL of 10% FBS-supplemented DMEM was added to each of the 24 wells of the Transwell plate (Corning), and 100 μL of Matrigel (Corning) was added to the cell culture inserts (8 μm). The plate was incubated in a humidified incubator at 37 °C with 5% CO_2 for an hour. 500 μL of SW480 WT, ELOF1 KO, NCSTN KO, OVOL2 KO or CAB39 KO cells were added to the Matrigel-coated inserts at 2×10^5 cells/ml in serum-free DMEM. The cells were incubated in the Matrigel-coated inserts for overnight in the incubator. The next day, medium was removed from the inserts and wells. The non-migrated cells on the upper side of the membrane were removed using a cotton swab and washed away with PBS. Cells remained on the membrane were fixed by submerging the membranes in the wells filled with ice cold methanol for 30 min and air-dried for 30 min. The membranes were then submerged in 750 μL 0.1 % crystal violet (Sigma) in wells for 30 min at room temperature to stain the cells before washed thoroughly with distilled water. After air-drying, membranes were submerged in 750 μL of 10 % acetic acid (VWR) with shaking until completely dissolving the stain. Optical density of the dissolved stain in each well was determined at 590 nm using a microplate reader.

Reverse transcription and quantitative real-time PCR.

After generating the knockouts or indicated treatments, cells were lysed in Tri-reagent (BioShop Canada #TSS120) and RNA extracted using the manufacturer's protocol. RNA concentration was quantified with Nanodrop1000 (Thermo Scientific) and 2 μg of RNA per sample was DNase I treated (ThermoFisher #AM2222). cDNA Reverse Transcription Kit (ThermoFisher #4368813) was used to make cDNA. Real-time PCR was performed using Power SYBR Green Master Mix on the 7900HT Fast Real-Time PCR system. Primer pairs are listed in Table 2. Analysis was done using the comparative cycle threshold (*CT*) method with all samples normalized to *GAPDH* expression.

Subcellular fractionation

Nuclei were prepared from SW480 cells, CACO-2 and RKO cells using a modification of the established protocol (Rosner et al., 2013) to accommodate for the high number of nuclei isolated. Throughout the isolation, the nuclei were processed in DNA LoBind® Tubes, (1.5ml, 15ml or 50ml), Catalog No. 0030122348, 0030122208 or 022431021 to obtain the most recovery possible. Briefly, every 2 x 10^7 cells were harvested by short trypsin digestion followed by FBS inactivation and washing in two rounds of PBS. Cells were incubated in nuclear isolation buffer (NIB; 10 mM Tris, 2 mM $MgCl_2$, 0.5 mM EDTA, pH 7.5) for 5 min at room temperature followed by 10 min on ice. The cells were disrupted by the addition of NP-40 to a final concentration of 1%. The mixture was centrifuged for 3 min at 500 x g to collect the nuclear fraction, followed by washing in NIB containing NP-40 prior to resuspension in buffer without additional NP-40. Nuclei were washed in a buffer made from the original NIB + NP-40 to a concentration of 0.1%. Next, nuclei were collected by centrifugation at 4 °C, 500 x g, for 3 min and resuspended in NIB. The integrity of purified nuclei was verified by (i) morphology and (ii) Western blot and (iii) immunofluorescence staining

Immunofluorescence microscopy

Cells were seeded onto poly-d-lysine-coated 8-well micro-slides (ibidi). The next day, media was removed, and the cells were washed twice with PBS and fixed with 100% ice-cold methanol for 5 min at −20 °C. After two more washes with PBS, cells were incubated with a blocking solution (3% BSA in PBS) at room temperature for 1 h. Anti-human CTNNB1 (***β***-Catenin (D10A8) XP® Rabbit mAb (Biotinylated)(13727S) and then Goat anti-Rabbit IgG (H+L) Secondary Antibody, FITC (Product # 31635) was used at a concentration of 4µg/mL in phosphate buffered saline containing 3 % BSA for 45 minutes at room primary antibodies were added at 1:50 and 1:500 for secondary antibodies in antibody dilution solution (1% FBS in PBS) and stained 1 hour at room temperature. Hoechst 33342 (ThermoFisher Scientific) was added at a 1:10,000 dilution in PBS for 5 min to label the nuclei. Finally, cells were washed 3x in PBS and imaged using a Leica SP5 700 confocal microscopes (Zeiss). For mean fluorescence intensity measurements, Hoechst staining intensity was used for colocalization analyses using Volocity software.

Genomic DNA extraction and Illumina sequencing

Genomic DNA was extracted from screen cell pellets using the Blood Maxi Genomic DNA Purification kit (Qiagen). Sequencing libraries were prepared by amplifying sgRNA inserts by a two-step PCR reaction using primers that include Illumina TruSeq adapters with i5 and i7 indices. The resulting libraries were subsequently sequenced on an Illumina HiSeq2500 as previously described (1). Each read was completed with standard primers for dual indexing with Rapid Run V1 reagents. The first 20 cycles of sequencing were dark cycles or base additions without imaging. The actual 26 bp read begins after the dark cycles and contains two index reads, reading the i7 first, followed by i5 sequences. The primers used for sequencing are provided in Supplementary Table 2.

Intracellular Flow cytometry and FACS

Throughout the sorting or isolation, the nuclei were processed in DNA LoBind® Tubes, DNA LoBind® (1.5ml, 15ml or 50ml), Catalog No. 0030122348, 0030122208 or 022431021 to obtain the most recovery possible. Cells were dissociated with 0.125% trypsin and washed once in flow buffer (PBS with 3% BSA). Nuclei were isolated as described above. Nuclei were fixed and permeabilized by submerging in ice cold (-20) Methanol for 30 min on ice. Then, the nuclei were washed 3x with (PBS +3% BSA). Antibody staining was carried out in flow buffer for 45 min at room temperature at 4 °C in the dark. Nuclei were washed for 3x to remove excess antibody and then incubated with FITC

secondary antibody for 20 min in the dark. Stained nuclei were washed three times with flow buffer. The following antibodies were used for these studies: anti- *β*-Catenin (D10A8) XP® Rabbit mAb (Biotinylated)(13727S) (1 µl per 2 x 10^6 nuclei in 100 µl) Goat anti-Rabbit IgG (H+L) Secondary Antibody, FITC (1:2000 ratio),: Stained nuclei were quantified using an LSRII flow cytometer (BD Biosciences and data were analyzed using FlowJo software (RRID: SCR_008520). MFI was defined as median fluorescence across the population and was displayed relative to wild-type cells in the same experiment. The gating strategy is outlined in Supporting Fig.8. Post FACS sorting, the nuclei from both the top 15% and bottom 15% were collected in a DNA Lo-bind tube. Next, the nuclei were centrifuged and proceeded to DNA extraction.

Western Blotting

Whole cell extracts were prepared as previously described (4). Briefly, cells or nuclei were collected with RIPA buffer (ThermoFisher) supplemented with protease inhibitor cocktail (Sigma). Proteins were separated on 4-15 % precast protein gels (Bio-Rad) and transferred onto PVDF membranes. Primary antibodies used were anti- *β*-Catenin (D10A8) XP® Rabbit mAb (Biotinylated)(13727S) anti- Lamin A/C (4C11) Mouse mAb (CST #4777) and anti-alpha Tubulin Rabbit Polyclonal antibody (Product # PA1-38814, 1:2500 dilution). Secondary antibodies were purchased from Thermofischer. Blots were visualized with chemiluminescent substrate (Thermo Scientific) using a ChemiDoc imaging system. Intensity of the protein bands were quantified using ImageJ.

Clonogenic assays

Cells were cultured and seeded into 6-well plates at a density of 1 × 10^5–2.5 × 10^5 cells per well, depending on growth rate, and were cultured in medium containing the indicated drugs for 2–4 days. Cells were fixed with 95% Methanol in PBS and stained with 0.1% crystal violet diluted in water. Cell confluence in each well was quantified using plate reader after dissolving the stained cells in 10% acetic acid. Optical density of the dissolved stain in each well was determined at 590 nm using a microplate reader.

References

1 Mair B, Aldridge PM, Atwal RS, Philpott D, Zhang M, Masud SN, et al. High-throughput genome-wide phenotypic screening via immunomagnetic cell sorting. Nat Biomed Eng. 2019;
2 Mezzadra R, Sun C, Jae LT, Gomez-Eerland R, De Vries E, Wu W, et al. Identification of CMTM6 and CMTM4 as PD-L1 protein regulators. Nature. 2017;
3. Li W, Xu H, Xiao T, Cong L, Love MI, Zhang F, et al. MAGeCK enables robust identification of essential genes from genome-scale CRISPR/Cas9 knockout screens. Genome Biol. 2014;
4. MacLeod G, Bozek DA, Rajakulendran N, Monteiro V, Ahmadi M, Steinhart Z, et al. Genome-Wide CRISPR-Cas9 Screens Expose Genetic Vulnerabilities and Mechanisms of Temozolomide Sensitivity in Glioblastoma Stem Cells. Cell Rep. 2019;
